# Retrospective study of glycemic variability, BMI, and blood pressure in diabetes patients in the Digital Twin Precision Treatment Program

**DOI:** 10.1038/s41598-021-94339-6

**Published:** 2021-07-21

**Authors:** Paramesh Shamanna, Mala Dharmalingam, Rakesh Sahay, Jahangir Mohammed, Maluk Mohamed, Terrence Poon, Nathan Kleinman, Mohamed Thajudeen

**Affiliations:** 1 Twin Health, Bangalore, Karnataka India; 2 Bangalore Endocrinology & Diabetes Research Centre, Bangalore, Karnataka India; 3 Department of Endocrinology, Osmania Medical College, Hyderabad, Telangana India; 4 Twin Health, Mountain View, CA USA; 5 Kleinman Analytic Solutions, LLC., Missouri City, TX USA

**Keywords:** Type 2 diabetes, Nutrition

## Abstract

The objective of this retrospective observational cohort study was to measure glycemic variability and reductions in body mass index (BMI), blood pressure (BP), and use of antihypertensive medications in type 2 diabetes (T2D) patients participating in the digital twin-enabled Twin Precision Treatment (TPT) Program. Study participants included 19 females and 45 males with T2D who chose to participate in the TPT Program and adhered to program protocols. Nine additional enrollees were excluded due to major program non-adherence. Enrollees were required to have adequate hepatic and renal function, no myocardial infarction, stroke, or angina ≤ 90 days before enrollment, and no history of ketoacidosis or major psychiatric disorders. The TPT program uses Digital Twin technology, machine learning algorithms, and precision nutrition to aid treatment of patients with T2D. Each study participant had ≥ 3 months of follow-up. Outcome measures included glucose percentage coefficient of variation (%CV), low blood glucose index (LBGI), high blood glucose index (HBGI), systolic and diastolic BP, number of antihypertensive medications, and BMI. Sixty-four patients participated in the program. Mean (± standard deviation) %CV, LBGI, and HBGI values were low (17.34 ± 4.35, 1.37 ± 1.37, and 2.13 ± 2.79, respectively) throughout the 90-day program. BMI decreased from 29.23 ± 5.83 at baseline to 27.43 ± 5.25 kg/m^2^. Systolic BP fell from 134.72 ± 17.73 to 124.58 ± 11.62 mm Hg. Diastolic BP decreased from 83.95 ± 10.20 to 80.33 ± 7.04 mm Hg. The percent of patients taking antihypertensive medications decreased from 35.9% at baseline to 4.7% at 90 days. During 90 days of the TPT Program, patients achieved low glycemic variability and significant reductions in BMI and BP. Antihypertensive medication use was eliminated in nearly all patients. Future research will focus on randomized case–control comparisons.

## Introduction

Type 2 diabetes is a chronic disease that typically leads to increased medication use, health risks, complications, and health care costs^1^. Care often focuses only on improving symptoms and slowing the progression of the disease^2^.

Glycemic variability has been shown to have a significant impact on the risk of diabetes complications. Glucose variability is responsible for the development and progression of diabetes complications by causing endothelial dysfunction through oxidative stress^3–5^. Glucose variability promotes hypoglycemic and hyperglycemic episodes that may increase the risk of diabetes complications and impair quality of life^6,7^. Glycemic variability has also been associated with cardiovascular autonomic neuropathy in type 2 diabetes patients^8^. In a review of ten type 2 diabetes studies, nine studies found that glucose variability was significantly associated with the development or progression of micro- and macrovascular complications^9^.

Several metrics are commonly used to measure glycemic variability. The percent coefficient of variation (%CV), standard deviation of blood glucose, continuous overall net glycemic action (CONGA), low blood glucose index (LBGI), and high blood glucose index (HBGI) have been used frequently to track glycemic variability in order to reduce hypoglycemic and hyperglycemic events^10–14^.

Insulin and certain oral hypoglycemic medications reduce glycemic variability^15,16^. Multiple studies have shown improvements in glycemic control from bariatric surgery, low-calorie diets, or carbohydrate restriction^2,17–19^. However, adverse events and high cost can limit the use of bariatric surgery^2^. Low-calorie diets (e.g. less than 800 kcal/day) to manage diabetes may be difficult to maintain over the long term^1^. Low carbohydrate studies showed improved glycemic control, but often were only small short-term trials, excluded subjects taking insulin, or were limited to morbidly obese patients^1,18–24^. None of the studies so far have combined long-term continuous glucose monitoring (CGM), artificial intelligence methods, and precision nutrition for long-term management of glycemic variability.

Virtual digital twins have recently been envisioned for the management of certain metabolic conditions^25^. The Twin Precision Treatment (TPT) Program uses CGM and detailed participant food intake data as inputs to a digital twin-enabled machine learning predictive model in the treatment of type 2 diabetes. The model provides precision nutrition guidance each day to the patient. Some results from the TPT Program have been reported previously^26^. In 64 patients with type 2 diabetes who enrolled and adhered to the TPT Program, mean (standard deviation) HbA1c decreased from 8.8% (2.2%) at baseline to 7.7% (1.6%) after 30 days and 6.9% (1.1%) after 60 and 90 days (all p < 0.0001 vs. baseline). Body weight decreased from 79.0 (16.2) kg at baseline to 74.2 (14.7) kg at 90 days (p < 0.0001). Fasting blood glucose changed from 151.2 (45.0) mg/dl at baseline to 129.1 (36.7) mg/dl at 90 days (p = 0.0001). By the first week of the program, most patients (55 of 64) had glucose time in range > 70%, and 57 of 64 had time in range > 70% at 90 days. The patients also had a significant decrease in insulin resistance and a significant increase in number of steps taken per day. Most patients were able to stop taking hypoglycemic medications^26^.

Given patients’ reductions in HbA1c, weight, fasting blood glucose, and use of hypoglycemic medications, our hypothesis was that patients in the TPT program would also have reduced glycemic variability, blood pressure, body mass index (BMI), and use of antihypertensive medications. The objective of the current study was to measure changes in glycemic variability, BMI, systolic and diastolic blood pressure, and antihypertensive medication use in patients using this novel TPT digital-twin approach to managing type 2 diabetes for 90 days.

## Methods

### Study design and patient population

This retrospective study followed 64 type 2 diabetes patients in India who chose to participate in the Twin Precision Treatment Program and adhered to program protocols. Each patient had at least 3 months of follow-up. The first patient enrolled in November 2018, and the patients completed the 3-month follow-up period by December 2019. To be included in the program, participants were required to have adequate hepatic and renal function (defined as aspartate transaminase/alanine transaminase ratio ≤ three times the upper limit of normal and serum creatinine ≤ 1.5 mg/dl or estimated glomerular filtration rate > 60 ml/min/1.73 m^2^). Patients with a history of ketoacidosis, major psychiatric disorders, or myocardial infarction, stroke, or angina within 90 days prior to enrollment were excluded. This study was approved by the Medisys Clinisearch Ethical Review Board. The study was performed in accordance with the Helsinki Declaration of 1964 and its later amendments. A written informed consent was obtained from all the subjects.

### TPT Program

The outpatient Twin Precision Treatment Program uses Whole Body Digital Twin technology, artificial intelligence and Internet of Things, to assess the patient’s unique metabolic impairment. Using body sensors and a mobile phone application (app), the platform collects data to track and analyse the body’s health signals and personalizes each patient’s treatment. The TPT platform uses artificial intelligence technologies (including rule-based expert systems, classical machine learning algorithms, and deep learning algorithms) to construct a Digital Twin model of each patient with longitudinal biological data from sensors, bloodwork, and nutritional data reported via the TPT mobile application. The Digital Twin is a dynamic digital representation of each patient’s specific metabolism, enabling personalized prediction of future health states for different interventions and selection of the ideal intervention for each patient. This includes recommendation of the best nutrition, exercise, and sleep interventions for treating chronic conditions like T2D.

During TPT Program enrollment, each patient’s vitals, clinical history, electrocardiogram and biothesiometry were assessed. Fasting blood draws were done in 30-day increments at baseline, 30 days, 60 days, and 90 days. The TPT Program was supported by assistance from health coaches. Upon receiving the initial blood test report, installation of the Twin mobile app, and activation of the sensors, patients began to receive nutritional inputs from the app and assisted by the health coach. Each patient was asked to wear a Fitbit Charge 2 sensor watch to continuously record heart rate, sleep parameters, step count, and other fitness parameters. Patients were asked to use a digital Bluetooth-enabled blood pressure meter (TAIDOC TD-3140) daily to record their blood pressure. Patients used a Powermax BCA-130 Bluetooth Smart Scale to measure their weight each morning after the first void of urine. Patients measured blood beta-hydroxybutyrate (BHB) levels with a finger prick each day. Continuous glucose monitoring (CGM) was performed to create Ambulatory Glucose Profiles throughout the study using an Abbott Libre Pro CGM Diabetes Sensor. This information was integrated into the TPT Program web-based software. All these data were transmitted to the software securely through a cellular network. The software access and biometric feedback sensor information were made available to the patients through the Twin app.

Patients were asked to record their food intake on the app. To see what drove glucose response to specific foods for each participant, machine learning algorithms analysed the macronutrients, micronutrients, and biota nutrients from the database. Several types of machine learning algorithms were used, including gradient boosted decision trees, deep learning neural networks, and long short-term memory models. For each participant, factors were analysed that were found to be associated with higher glycemic response. Then participants were provided with specific food recommendations to avoid glucose spikes. Using machine learning algorithms and data fusion techniques that incorporated the continuous glucose monitor data, blood glucose values of the participants were predicted. Participants logged each food item and its quantity from every meal by selecting it from a database of more than 2000 foods together with full nutritional values. This information was based on the U.S. Department of Agriculture FoodData Central database^27^ and was further improved and expanded with additional items from certified sources^28^. The machine learning algorithm combined these multi-dimensional data to predict postprandial glucose response (PPGR) for each person. When the algorithm detected foods that led to high glucose area under the curve (AUC) values, it would then suggest foods with lower AUCs for that patient. Because, diet is a central determinant of blood glucose levels, it is essential to make food choices that induce normal PPGR in order to achieve normal glucose levels.

The goal of the Twin program is to provide the optimal combination of macronutrients, micronutrients and biota nutrients, while guiding each patient to eat foods that do not produce glucose spikes and to avoid foods that cause blood glucose spikes. The program placed no limit on calorie consumption, and patients were allowed to eat to satiety. Trained coaches provided nutritional assistance through telephone calls to assist in following the guidance from the app.

When titrating patient medications, physicians followed medication guidelines, taking into account CGM values and patient characteristics. These medication changes were suggested by the Twin platform and were approved or altered by the physician. Patients’ insulin doses were adjusted based on their daily average blood glucose levels. Metformin and DPP-4 inhibitors were provided and titrated over time as blood sugar levels improved. Further medication management by the physician was based on customized guidelines. All patients’ symptoms were analysed and managed by the physicians.

### Outcome measures

Patient age, gender, and duration of diabetes were recorded at enrollment. BMI (kg/m^2^) and systolic and diastolic blood pressure (mm Hg) were assessed at baseline (program enrollment) and at 30, 60, and 90 days after enrollment. Fasting insulin (mIU/l) and the percent of patients taking antihypertensive medications were calculated at baseline and at 90 days of program participation. Using 96 daily blood glucose readings from each patient’s continuous glucose monitor, daily glycemic variability values for percent coefficient of variation (%CV), low blood glucose index (LBGI), and high blood glucose index (HBGI) were calculated from published formulas^7,29^ and averaged over the seven days ending at day 30, day 60, and day 90 and also averaged over the entire 90-day period. Values of %CV were also available for the first day of program participation. Values of the standard deviation of blood glucose were calculated on the first day of participation and during the seven days ending at day 90. CONGA values were calculated on the first and 90th days of participation. Lipid profile values were assessed at baseline and at 30 and 90 days.

### Statistical analysis

Age, duration of diabetes, BMI, and blood pressure were described using means and standard deviations. Mean, median, minimum, and maximum values of glycemic %CV, LBGI, and HBGI were assessed. Percentages were used to report means of binary variables. Changes in mean values of continuous variables from baseline to 30, 60, and 90 days after enrollment were assessed using paired t-tests. Changes in average values of binary variables were assessed using McNemar’s chi-squared test. Tests of changes in values excluded the few patients with missing values.

### Other assistance

The authors would like to acknowledge our doctors Dr. Mohammed Abul Hassan and Dr. Rajamohan for using TPT with their patients. We offer our appreciation and acknowledgement to the operation team headed by Mr. Hanif Syed and his team, Ms. Monika Nanda and Ms. Abinaya, who offered necessary support in handling sensors, blood investigation coordination and health coaching. We would also like to acknowledge the engineering and product design team for making all necessary technology support in offering a friction-free best patient experience.

### Compliance with ethics guidelines

This study was approved by the Medisys Clinisearch Ethical Review Board. The study was performed in accordance with the Helsinki Declaration of 1964 and its later amendments. A written informed consent was obtained from all the subjects.

### Study participants

The authors would like to express thanks to the participants of the study.

## Results

Seventy-three patients enrolled in the TPT Program. Nine of these patients were removed from the analysis because of significant program non-adherence. The 64 patients who enrolled in and adhered to the TPT Program had a mean (standard deviation, SD) age of 52.44 (9.96) years at enrollment. Nineteen of the patients were female (29.69%). The patients had had diabetes for 8.43 (6.52) years at enrollment, on average (Table 1).

**Table 1:** Patient population descriptive information at enrollment.

Number of patients	64
Age, mean years (SD)	52.44 (9.96)
Percent female	29.69%
Duration of diabetes, mean years (SD)	8.43 (6.52)

*SD* standard deviation.

**Figure 1:**
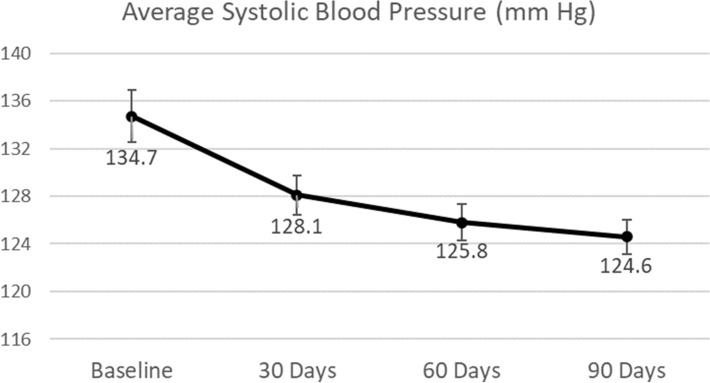
Changes in systolic blood pressure. Vertical bars represent standard errors.

**Figure 2:**
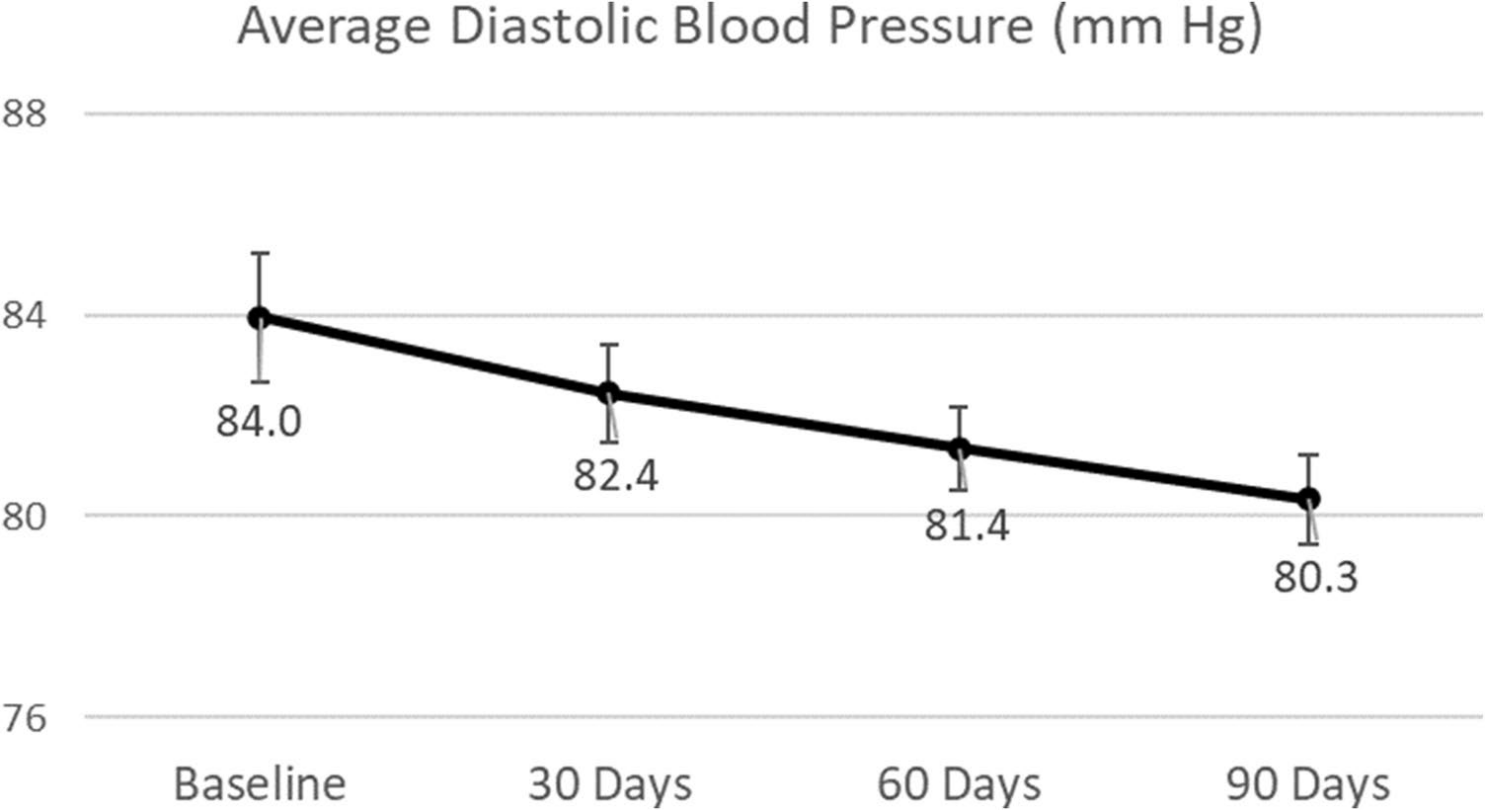
Changes in diastolic blood pressure. Vertical bars represent standard errors.

The patients’ mean (SD) BMI (kg/m^2^) decreased significantly over the study period, from 29.23 (5.83) at baseline to 28.12 (5.50) at 30 days, 27.87 (5.35) at 60 days, and 27.43 (5.25) at 90 days (all p < 0.0001 vs. baseline) (Table 2). Systolic blood pressure (mm Hg) fell from 134.72 (17.73) at baseline to 128.10 (12.96) at 30 days (p = 0.0002), to 125.79 (12.03) at 60 days (p < 0.0001), and to 124.58 (11.62) at 90 days (p < 0.0001) (Fig. 1). Diastolic blood pressure (mm Hg) decreased from 83.95 (10.20) at baseline to 82.44 (7.61) at 30 days (p = 0.1492), to 81.35 (6.59) at 60 days (p = 0.0117), and to 80.33 (7.04) at 90 days (p = 0.0020) (Fig. 2). Additionally, fasting insulin levels (mIU/l) changed from 27.13 (27.88) at baseline to 10.11 (7.93) at 90 days (p < 0.0001).

**Table 2:** Anthropometric and metabolic metrics at baseline and the first 3 months.

	Baseline			30 days				60 days				90 days			
	N	Mean	Standard deviation	N	Mean	Standard deviation	p-value vs. Baseline	N	Mean	Standard deviation	p-value vs. Baseline	N	Mean	Standard deviation	p-value vs. Baseline
Body mass index (kg/m^2^)	64	29.23	5.83	64	28.12	5.50	< 0.0001	63	27.87	5.35	< 0.0001	64	27.43	5.25	< 0.0001
Systolic blood pressure (mm Hg)	64	134.72	17.73	63	128.10	12.96	0.0002	63	125.79	12.03	< 0.0001	64	124.58	11.62	< 0.0001
Diastolic blood pressure (mm Hg)	64	83.95	10.20	63	82.44	7.61	0.1492	63	81.35	6.59	0.0117	64	80.33	7.04	0.0020

Twenty-three patients (35.94%) were taking antihypertensive medications at baseline, but only 3 patients (4.69%) required antihypertensive medications after 90 days of the program (p < 0.0001).

**Figure 3:**
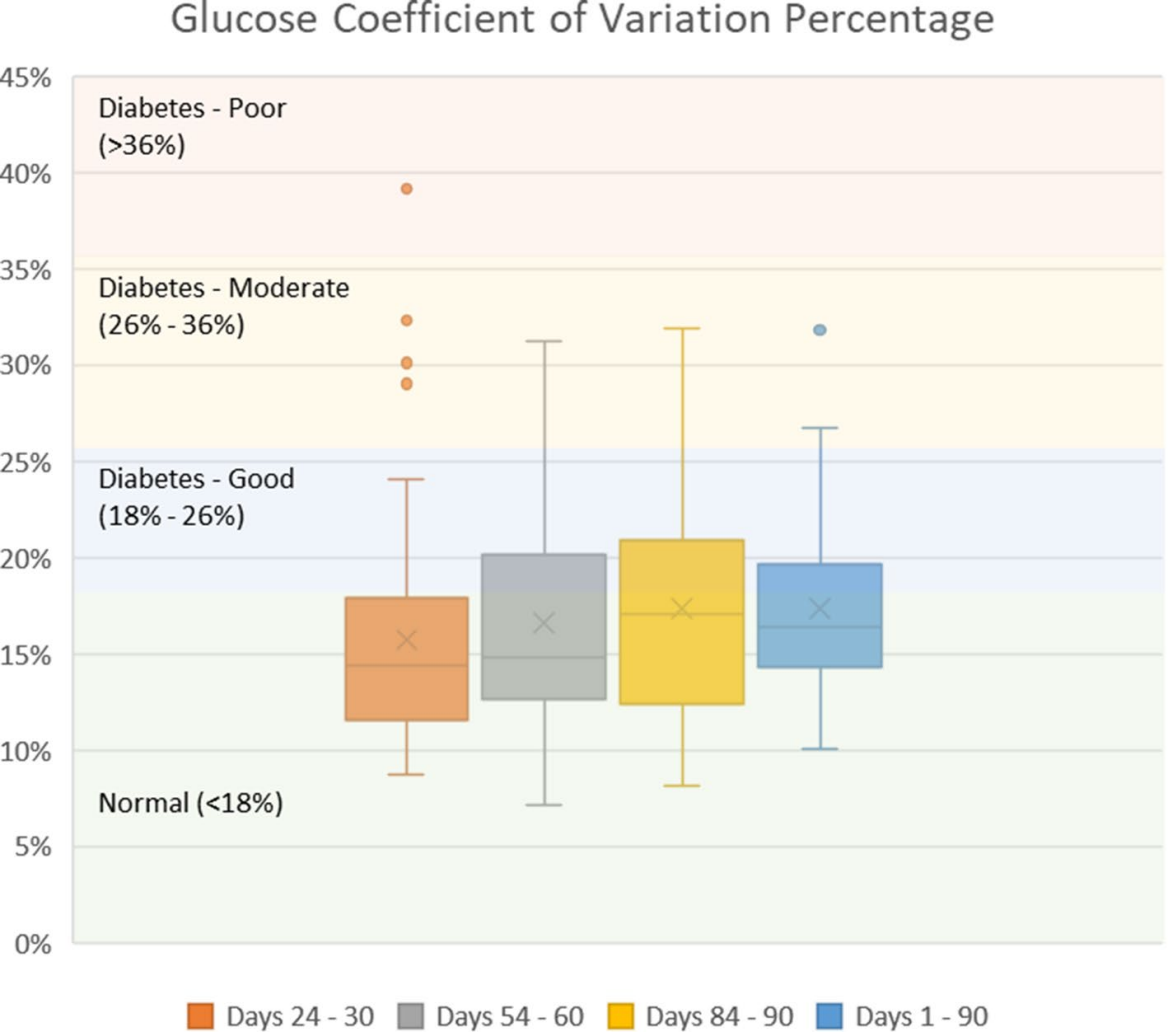
Glucose percent coefficient of variation during the first 90 days of program participation.

On the first day of program participation, the mean (SD) glucose variability (%CV) was 20.58% (10.22). Mean and median glucose variability were maintained < 18% during the study. Mean %CV (SD) was 15.75% (6.10) during days 24 to 30 (p = 0.0006 vs first day), 16.62% (5.96) during days 54 to 60 (p = 0.0025), and 17.37% (6.00) during days 84 to 90 of the program (p = 0.0276). Similarly, over all 90 days of the study the average %CV was 17.34% (4.36) (p = 0.0102) (Fig. 3, Table 3).

**Table 3:** Glycemic variability metrics.

Glycemic variability metric	Threshold	Day 24–30			Day 54–60			Day 84–90			Day 1–90		
		Mean	Standard deviation	p-value vs. threshold	Mean	Standard deviation	p-value vs. threshold	Mean	Standard deviation	p-value vs. threshold	Mean	Standard deviation	p-value vs. threshold
Coefficient of variation percentage (%CV)	< 18%	15.75	6.10	0.0044	16.62	5.96	0.0684	17.37	6.00	0.4054	17.34	4.35	0.2304
Low blood glucose index (LBGI)	Low (< 2.5)	1.77	2.59	0.0279	1.14	1.55	0.0000	1.21	1.76	0.0000	1.37	1.37	0.0000
High blood glucose index (HBGI)	Low (< 4.5)	1.62	2.65	0.0000	1.89	3.46	0.0000	2.14	4.52	0.0001	2.13	2.79	0.0000

Mean (SD, N) standard deviation of blood glucose (mg/dl) on the first day of program participation was 28.59 (14.32, 62). This decreased to 20.96 (8.71, 64) during days 84 to 90 (p < 0.0001). The mean (SD, N) CONGA value (mmol/l) on the first day of program participation was 7.38 (3.39, 63), and this decreased to 6.39 (1.98, 56) on the 90th day (p = 0.0254).

**Figure 4:**
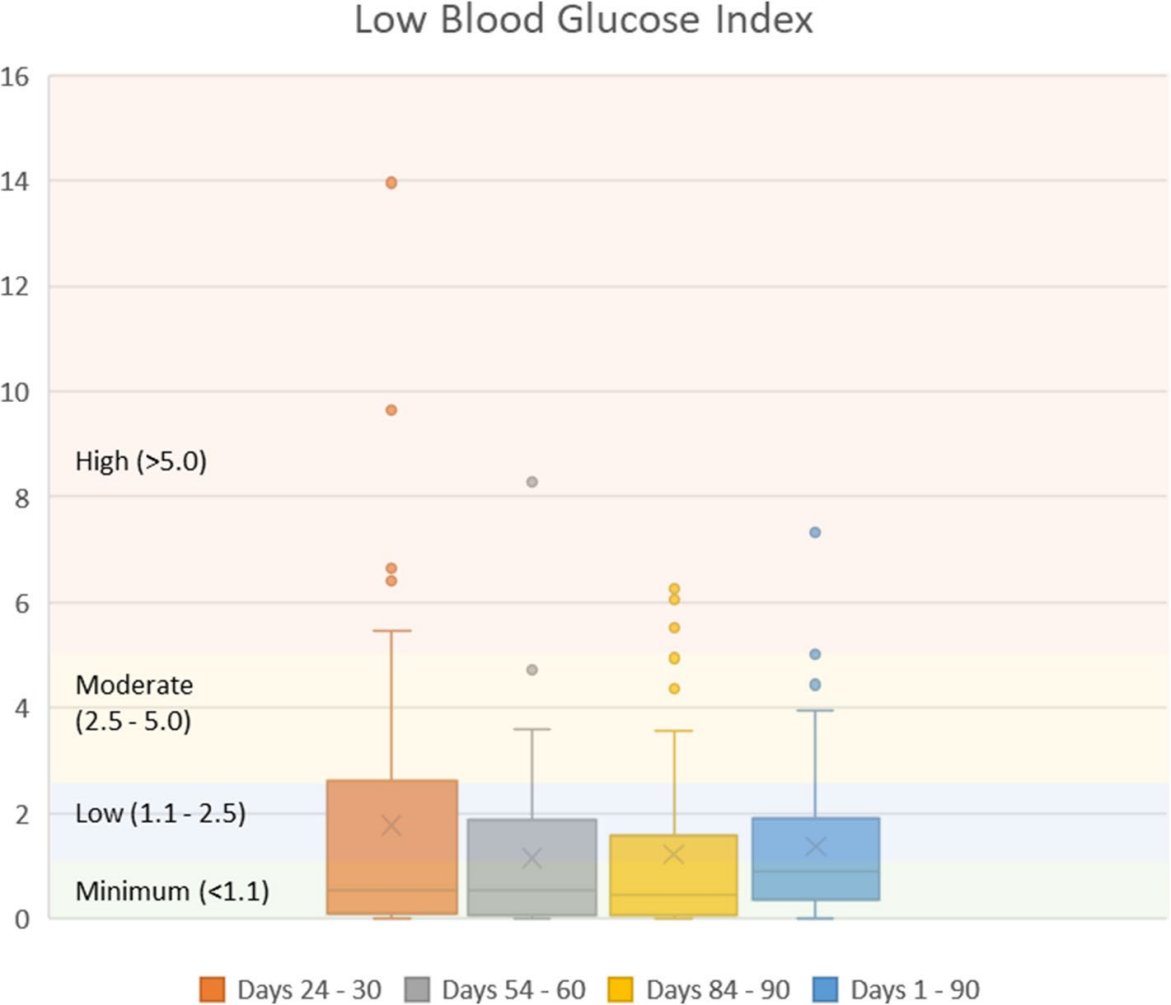
Low blood glucose index during the first 90 days of program participation.

**Figure 5:**
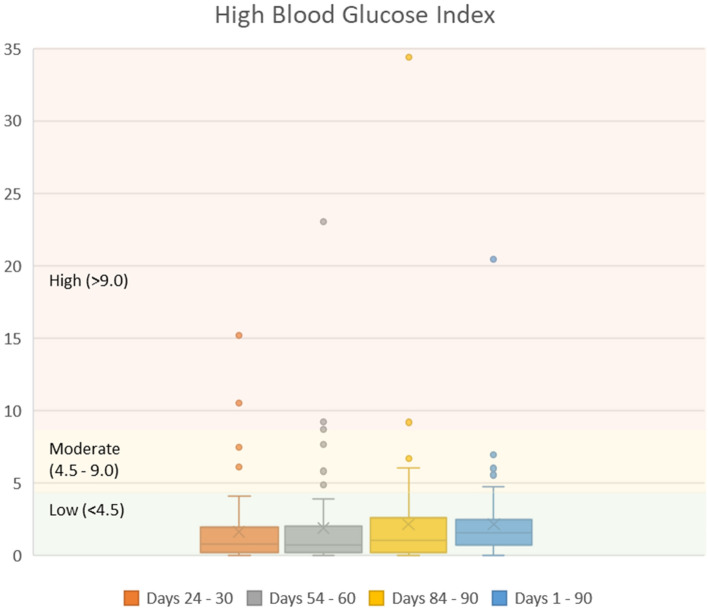
High blood glucose index during the first 90 days of program participation.

Both mean and 75th percentile values of LBGI were within the low^13–15^ LBGI threshold level (≤ 2.5) during the study. Mean (SD) LBGI values during 24 to 30 days, 54 to 60 days, and 84 to 90 days were 1.77 (2.59), 1.14 (1.55), and 1.21 (1.76), respectively. Over all 90 days of the program, the average LBGI value was 1.37 (1.37) (Fig. 4). A high percentage of patients had HBGI values within the low^13,15^ HBGI threshold level (< 4.5) during the study: 92.2%, 89.1%, and 87.5% during days 24 to 30, 54 to 60, and 84 to 90, respectively. Over days 1 to 90 of the study period, 90.6% of patients had HBGI < 4.5 (Fig. 5).

Mean (SD) high-density lipoprotein (HDL) cholesterol values increased from 42.9 (10.5) at baseline to 44.1 (14.2) at 30 days and 48.0 (12.2) at 90 days. Low-density lipoprotein values changed from 103.7 (33.9) at baseline to 120.2 (39.9) at 30 days and 117.5 (47.9) at 90 days. Non-HDL cholesterol was 138.1 (37.5) at baseline, 154.9 (41.9) at 30 days, and 144.1 (52.0) at 90 days. Total cholesterol changed from 181.0 (39.2) at baseline to 201.0 (48.0) at 30 days and 192.1 (51.0) at 90 days. Triglycerides decreased from 186.2 (103.8) at baseline to 157.7 (92.9) at 30 days and 135.1 (77.5) at 90 days. Finally, the ratio of triglycerides/HDL changed from 4.7 (3.4) at baseline to 4.9 (7.4) at 30 days and 3.1 (2.1) at 90 days.

## Discussion

The CGM, food intake data, Internet of Things technology, and machine learning algorithms in the Twin Precision Treatment Program optimized combinations of nutrients and provided nutritional guidance to type 2 diabetes patients that helped them consume foods that do not produce glucose spikes and avoid foods that cause blood glucose spikes.

The program participants in the current study reduced mean systolic blood pressure by 7.5% (p < 0.0001) and reduced diastolic blood pressure by 4.3% (p = 0.002) from baseline over the course of the 90-day study period. The TPT Program recommended patient-specific meal plans that depended on the likes and dislikes of the patient and that were balanced across macro, micro, and biota nutrients to reduce glucotoxicity and lipotoxicity. This helped to heal inflammation and may be a reason for the improved blood pressure. Patients were also provided with supplements to ensure sufficient micronutrients were consumed. Nutritional, activity, and sleep counselling were provided by trained health coaches through the app and via telephone. Additionally, the digital twin technology enabled precise management of nutrition, activity, and sleep and helped the coach focus on the most important lifestyle variables for that patient for the improvement of blood pressure. Daily home blood pressure monitoring was done by the patient, and measurements were transmitted through Bluetooth-enabled equipment to the patient’s mobile app and to the platform and were used by the coaches to measure the impact of these interventions. Hence a feedback loop was established which enabled the efficient reduction of blood pressure.

Improvements over the 90 days were also seen in BMI (6.2% reduction, p < 0.0001). BMI reductions may have stemmed from the nutritional interventions, increases in physical activity (mean (SD) steps taken per day increased from 4677.4 (2804.9) at baseline to 7004.1 (3999.0) at 90 days)^26^, the 63% reduction in fasting insulin level, and the reduction in average homeostatic model assessment of insulin resistance (HOMA-IR) from 7.4 (3.5) at baseline to 3.1 (2.5) at 30 days and 3.2 (2.8) at 90 days^26^. Additionally, the percent of patients taking antihypertensive medications decreased from 35.9 to 4.7% (p < 0.0001). For most patients, glycemic variability was stabilized during the first week of the program, and %CV, LBGI, and HBGI were maintained within normal or low thresholds over the 90 days.

Hypertension is found in more than half of patients with diabetes. Hypertension increases diabetes patients’ risk of micro- and macrovascular disease and chronic kidney disease and increases costs. Diabetes and hypertension both have insulin resistance in common, and both improve with lifestyle intervention. Controlling blood pressure in patients with diabetes prevents and delays micro- and macrovascular complications^30,31^. Specifically, among patients with type 2 diabetes, each 10-mm Hg reduction in blood pressure has been associated with improved mortality, reductions in cardiovascular events, coronary heart disease, stroke, albuminuria, and retinopathy^32^.

The literature suggests that blood pressure goals of systolic/diastolic blood pressure < 130/80 mm Hg are rarely attained in patients with diabetes and that treatments employing at least two medications are needed for most patients^31^. In the present study, patients reduced their mean systolic blood pressure from 134.7 to 124.6 mm Hg and reduced mean diastolic blood pressure from 83.9 to 80.3 mm Hg at 90 days, with less than 5% of patients taking antihypertensive medication by the end of the study. After 10 weeks of intensive nutrition, behavioural counselling, digital coaching, and medication management, McKenzie et al. found similar blood pressure reductions in a much more obese population (a reduction from 132 to 125 mm Hg in systolic blood pressure and a reduction from 82 to 78 mm Hg in diastolic blood pressure)^1^.

Monitoring BMI in patients with type 2 diabetes is important, as increased BMI is associated with decreased life expectancy^33^. Prior studies have reported some improvements in BMI after certain types of intervention. In the intensive nutritional study by McKenzie et al., BMI decreased 7.2% in a population of morbidly obese patients with type 2 diabetes^1^. After 12 weeks of low-carbohydrate or low-glycemic diets, type 2 diabetes patients in another study^22^ reduced BMI from 37.8 to 34.4 (9.0%) and from 37.9 to 36.5 (3.7%), respectively. Over a similar time period, the present study found a 6.2% reduction in the BMI of patients whose initial mean BMI was 29.

Limited interventional studies were found that reported %CV, LBGI, and HBGI results. In a study by Ohara et al. of normal treatment of type 2 diabetes patients with mean (SD) duration of diabetes of 11.6 (9.2) years, mean %CV began at 24.3% and decreased to 21.7% at 24 weeks^34^. In patients with mean duration of diabetes of 9.0 (6.0) years, Inzucchi et al. reported an increase in %CV from 21.0 to 25.7% and a decrease in HBGI from 15.4 to 5.3 in a 24-week study of type 2 diabetes patients who received an intensification of their insulin treatment^35^. In patients with duration of diabetes of 8.43 (6.52) years who participated in the TPT Program, the current study found that %CV over the 90-day program was substantially lower at 17.3%.

In a study of 549 patients with type 2 diabetes taking insulin or oral hypoglycemic medications, McCall et al. found that mean daily LBGI and HBGI values over weeks 8 to 26 of the study were approximately 0.5 (minimal range) and 5.5 (moderate range), respectively^15^. In the present study, mean LBGI and HBGI over days 1 to 90 were 1.37 (low range) and 2.13 (low range), respectively, and most patients were able to eliminate hypoglycemic medication use over that time^26^. As reported previously, all 12 patients who had been taking insulin were able to discontinue its use, two-thirds of the patients on metformin discontinued taking metformin, and almost every patient on other oral hypoglycemic medications was able to stop taking them^26^. Other metrics for this population, such as HbA1c, fasting glucose, time in range, and weight, were reported previously^26^.

No cases of diabetic ketoacidosis and no episodes of symptomatic hypoglycemia were observed in the current study. Additionally, no new cases of gout were reported. The overall average patient-reported program satisfaction score was 4.4 out of 5.

The strengths of this study include significant reductions in BMI and blood pressure and the maintenance of %CV, LBGI, and HBGI below low thresholds, while eliminating antihypertensive and hypoglycemic medication use in nearly all patients. The out-patient nutritional intervention employed home-cooked foods, making the intervention sustainable. Accurate assessment of nutrition was aided by the patients’ app-based logging of food.

Limitations of this study include its smaller study population, retrospective nature, and the lack of a control group. Additionally, increasing the length of the program will be a goal of subsequent studies.

## Conclusion

Following 90 days of the TPT Program, patients achieved low glycemic variability and significant reductions in BMI and blood pressure. Antihypertensive medication use was eliminated in nearly all patients. Future results will examine the long-term effectiveness and sustainability of the program.

## Data Availability

The datasets analysed during the current study are available from the corresponding author on reasonable request.
